# Tumor-induced suppressive CD8+ T cells: implications for cancer immunotherapy

**DOI:** 10.1186/2051-1426-1-S1-P171

**Published:** 2013-11-07

**Authors:** Lukas W Pfannenstiel, Brian R Gastman

**Affiliations:** 1 Immunology, Cleveland Clinic, Cleveland, OH, USA

## 

The immune system has the potential to be a powerful tool to destroy tumors; however despite ample evidence of endogenous anti-tumor immune responses in many patients, as well as years of immunotherapy development, truly effective immune-based therapies remain out of reach. We have previously shown that co-incubation of normal human T cells with various tumor lines can induce dysfunctional changes in the T cells characterized by the loss of CD27/CD28 expression, hypo-proliferation upon activation, and the gain of suppressive function in vitro. We also found that this process could be inhibited by IL-7 signaling, primarily through PI3k/AKT signaling, and enhancing the expression of the pro-survival molecule Mcl-1. In the current study, we use a mouse model of HPV+ head and neck cancer to show that the process of tumor-induced dysfunction also induces the expression of PD-1 in both human and mouse T cells, and that tumor-exposed mouse T cells are also capable of suppressive function. We further show that the tumor microenvironment induces large numbers of PD-1+ CD8+ T cells that are also positive for other negative regulators of T cell function including Tim-3, and that these cells are also suppressive ex vivo. Ongoing work will establish whether blockade of PD-1 and Tim-3, as well as in vivo systemic treatment with IL-7 concurrent with adoptive transfer of tumor-specific T cells is able to resist the induction and function of dysfunctional, suppressive T cells in a manner similar to previous in vitro studies. Further work will also evaluate the role of Mcl-1 expression in generation of dysfunctional CD8 T cells in vivo.

